# Pharmacokinetics and tissue distribution analysis of ginsenoside Rh_3_ in rats using a novel LC-MS/MS quantification strategy

**DOI:** 10.3389/fphar.2025.1582644

**Published:** 2025-07-10

**Authors:** Cong Hu, Yuheng Wang, Yu Liu, Xiaojing Wang, Peifang Song, Hong Ma, Ling Yang

**Affiliations:** ^1^ Key Laboratory of Basic Pharmacology of Ministry of Education and Joint International Research Laboratory of Ethnomedicine of Ministry of Education, Zunyi Medical University, Zunyi, China; ^2^ Institute of Interdisciplinary Integrative Medicine Research, Shanghai University of Traditional Chinese Medicine, Shanghai, China; ^3^ Department of Neurology, The First Affiliated Hospital, Dalian Medical University, Dalian, China

**Keywords:** GRh_3_, pharmacokinetics, tissue distribution, blood-brain barrier, LC-MS/MS

## Abstract

**Introduction:**

Ginsenoside Rh3 (GRh_3_), a rare ginsenoside, demonstrates diverse pharmacological activities *in vitro*; however, the lack of pharmacokinetic and tissue distribution data has limited its translation to *in vivo* applications. This study aimed to develop and validate a novel liquid chromatography-tandem mass spectrometry (LC-MS/MS) method for quantifying GRh_3_ in rat biological matrices and to characterize its pharmacokinetic profile and tissue distribution following oral administration.

**Methods:**

A validated LC-MS/MS method was established for the quantification of GRh_3_ in rat plasma and tissues. Male Sprague-Dawley rats received an oral dose of GRh_3_ (100 mg/kg), and plasma samples were collected up to 72 h post-dose for pharmacokinetic analysis. Tissue samples (intestine, stomach, liver, brain, etc.) were collected at the time corresponding to the maximum plasma concentration for distribution analysis.

**Results:**

The LC-MS/MS method showed excellent precision, accuracy, and extraction recovery (≥ 85%), with minimal matrix effects. GRh_3_ exhibited a prolonged elimination half-life (14.7 ± 1.7 h), a low clearance rate (13.0 ± 3.8 L/h/kg), and a high volume of distribution (280.4 ± 109.3 L/kg). Tissue distribution analysis revealed the highest GRh_3_ concentrations in the intestine (15445.2 ng/g), followed by the stomach (2906.7 ng/g) and liver (1930.8 ng/g). Notably, GRh_3_ was able to cross the blood-brain barrier, with significant accumulation observed in the hippocampus (520.0 ng/g).

**Discussion:**

The prolonged elimination and extensive tissue distribution of GRh_3_, particularly its ability to penetrate the brain, indicate potential therapeutic benefits or neurotoxic risks involving the central nervous system. The mechanism underlying its blood-brain barrier permeability warrants further investigation, potentially involving transporter-mediated uptake or modulation of barrier integrity. These findings provide a foundation for optimizing GRh_3_ dosing regimens and guiding future preclinical studies.

## 1 Introduction

Ginseng (*Panax ginseng* C. A. Meyer) is a quintessential traditional medicinal plant. It has held pivotal roles in both the traditional Chinese medicine theory of “invigorating qi for relieving desertion” and modern medical systems, owing to its multifaceted therapeutic properties (Yun, 2001). As one of the most commercially valuable herbs globally, *ginseng* is also widely utilized in dietary supplements, functional foods, and alternative medicine (Baeg and So, 2013; Eom et al., 2017). Ginsenosides, characterized by their extensive pharmacological activities, are deemed the key bioactive compounds in ginseng (Hao et al., 2025). These can be classified into two major categories, prototype ginsenosides and rare ginsenosides, based on their natural abundance and the number of glycosyl substitutions (Gan et al., 2024). Among these, rare ginsenosides, which undergo structural modifications such as deglycosylation or hydroxylation, exhibit enhanced multi-target activity. These modifications contribute to their significant advantages in antitumor effects, neuroprotective effects, metabolic regulation, and organ-protective functions (Shah et al., 2023; Shan et al., 2023; Shang et al., 2025; Zare-Zardini et al., 2024).

Ginsenoside Rh_3_ (GRh_3_), as a secondary metabolite of ginsenoside Rg_5_, is an important member of the rare ginsenoside family (Lee et al., 2015). Recent studies have progressively revealed its multifaceted pharmacological effects. Experimental evidence demonstrates that GRh_3_ protects endometrial cells against oxygen-glucose deprivation-reperfusion (OGDR)-induced oxidative damage by activating the Nrf2 signaling pathway, and attenuating reactive oxygen species (ROS) generation and lipid peroxidation while preserving mitochondrial membrane potential stability (Wang et al., 2020). In metabolic regulation, GRh_3_ exerts systemic protective effects on hepatic function by modulating critical targets including epidermal growth factor receptor (EGFR), steroid receptor coactivator (SRC) and mitogen activated protein kinase (MAPK) 1 to ameliorate hepatic insulin resistance, while concurrently influencing forkhead box O (FOXO), peroxisome proliferator activated receptor (PPAR), and interleukin-17 (IL-17) signaling pathways (Wang et al., 2024). In the neuroprotective domain, GRh_3_ significantly alleviates memory dysfunction by upregulating hippocampal brain-derived neurotrophic factor (BDNF) expression and enhancing cAMP response element-binding protein (CREB) phosphorylation (Wu et al., 2023). Besides, GRh_3_ exhibits substantial anticancer potential through induction of pyroptosis and ferroptosis in colorectal cancer cells via the Stat3/p53/NRF2 axis (Kim et al., 2013).

Although numerous *in vitro* studies have confirmed the multifaceted pharmacological effects of GRh_3_, the absence of comprehensive data on its pharmacokinetics (PK) and tissue distribution has hindered the effective translation of these pharmacological findings into *in vivo* efficacy research. To date, only one study has reported the plasma PK profile of GRh_3_ in rats (Yang et al., 2022). However, the extraction recovery rate of the analytical method used in that study failed to meet bioanalytical regulatory standards (EMA, 2011; FDA, 2018), and the short sampling duration (with the last time-point blood concentration exceeding 10% of C_max_) in PK analysis compromised the accuracy of PK parameters (CDE, 2005). These limitations necessitate the redevelopment of a validated analytical method and subsequent reinvestigation of GRh_3_’s PK characteristics. Furthermore, the tissue distribution of GRh_3_ has not been investigated, resulting a significant gap in our understanding. Both PK and tissue distribution data are essential for advancing GRh_3_ development in pharmacodynamic exposure correlation analysis, toxicity risk assessment, and optimization of dosing strategies.

To address these challenges, this study successfully developed and validated a liquid chromatography-tandem mass spectrometry (LC-MS/MS) method for detecting GRh_3_ concentration in rat plasma and tissues. Using this method, a comprehensive investigation was conducted on the PK and tissue distribution of GRh_3_ following oral administration in rats. This work provides a critical foundation for the *in vivo* research and pharmaceutical development of GRh_3_.

## 2 Materials and methods

### 2.1 Chemicals and reagents

GRh_3_ and GRh_4_ (internal standard, IS) were obtained from Alfa Biotechnology Co., Ltd. (Chengdu, China). Both compounds had a purity greater than 98% and were of analytical grade. Acetonitrile and formic acid (HPLC grade) were obtained from Merck (Darmstadt, Germany). Ultrapure water was generated using a Millipore water purification system (Billerica, United States). Isoflurane was obtained from RWD Life Science Co., Ltd. (Shenzhen, China).

### 2.2 Animals

Male Sprague-Dawley rats (SPF grade, 200 ± 20 g) were procured from Charles River Laboratories (Zhejiang, China). The rats were acclimatized for 1 week under a 12 h light-dark cycle and were fasted for 12 h prior to GRh_3_ administration while being allowed free access to water. The study was approved by the Animal Experimentation Committee of Zunyi Medical University (Approval No. ZMU21-2403-453).

### 2.3 Pharmacokinetics experiment

Seven male Sprague-Dawley rats were orally administered GRh_3_ (100 mg/kg) suspended in 0.5% carboxymethyl cellulose sodium (CMC-Na). Blood samples were collected from the orbital sinus at the following time points: 0, 0.083, 0.249, 0.498, 0.747, 1, 2, 4, 6, 8, 12, 24, 36, 48, 60, and 72 h post-dose into polypropylene tubes containing K_2_EDTA. The samples were subsequently centrifuged at 3000 × *g* for 20 min at 4°C, and the supernatant (plasma) was collected for PK analysis.

### 2.4 Tissue distribution experiment

Seven male Sprague-Dawley rats were orally administered GRh_3_ (100 mg/kg) suspended in 0.5% CMC-Na. At T_max_ (the time corresponding to maximum plasma GRh_3_ concentration), the rats were euthanized and perfused with physiological saline via cardiac perfusion to minimize blood contamination in tissue samples. Subsequently, various tissues, including the liver, intestine, stomach, kidney, lung, heart, spleen, hippocampus, cerebral cortex, and brainstem were excised. Among these, the brain tissue sampling protocol followed previously published methods (Aboghazleh et al., 2024): target regions, including the cortex, hippocampus, and brainstem, were precisely dissected according to standard anatomical landmarks on a low-temperature dissection platform. To avoid cross-contamination between different brain regions, independent surgical instruments were used for each region, and all tools were disinfected and cleaned before and after sampling. The excised tissues were weighed and homogenized in PBS (0.1 M, pH 7.4) at 4°C using an automated tissue homogenizer (Shanghai Jingxin Industrial Development Co., Ltd., Shanghai, China). Tissue homogenates were prepared at a final concentration of 0.2 g/mL.

### 2.5 Preparation of calibration and quality control samples

Stock solutions of GRh_3_ and IS were prepared at 2.0 mg/mL in acetonitrile. The GRh_3_ stock solution was serially diluted with acetonitrile to produce standard calibration and quality control (QC) working solutions. 200 ng/mL IS working solution was obtained by diluting the IS stock solution with acetonitrile. 190 μL of blank rat plasma or blank liver homogenate was spiked with 10.0 μL GRh_3_ working solution to acquire 25 ng/mL (LLOQ), 50 ng/mL, 125 ng/mL, 250 ng/mL, 500 ng/mL, 2,000 ng/mL, 4,000 ng/mL, and 5000 ng/mL (ULOQ) for standard calibration samples, and 25 ng/mL (LLOQ QC), 100 ng/mL (LQC), 400 ng/mL (MQC), and 3000 ng/mL (HQC) for the QC samples.

### 2.6 Sample processing

40 μL of calibration samples, QC samples, or test samples were mixed with 160 μL of the IS working solution (200 ng/mL) and vortexed. The mixture was then centrifuged at 12,000 × *g* for 10 min at 4°C. Subsequently, 100 μL of the supernatant was collected and mixed with an equal volume of purified water. A 10 μL aliquot of this mixture was then injected into the LC-MS/MS system for analysis.

### 2.7 LC-MS/MS analysis

Sample analysis was conducted using an LC-MS/MS system, which consisted of a Shimadzu LC 20A liquid chromatograph (Kyoto, Japan) coupled with an Applied Biosystems Sciex Qtrap 4500 mass spectrometer (Massachusetts, United States). Chromatographic separation was performed on a Shimadzu Shim-pack GIST-HP C18 column (2.1 mm × 50 mm, 3 μm). The mobile phase consisted of (A) 0.1% formic acid in water and (B) acetonitrile. The gradient elution program was as follows: 0.0–1.0 min, 30%–98% B; 1.0–3.5 min, 98% B; 3.5–3.6 min, 98%–30% B; 3.6–5.0 min, 30% B, with a flow rate of 0.4 mL/min.

Mass spectrometric analysis was conducted in negative ion mode using multiple reaction monitoring (MRM). The ion source temperature was set to 550°C, with an ion spray voltage of −4500 V. Nebulizer gas (GAS1) and auxiliary heating gas (GAS2) were both set to 60 psi, while the curtain gas was set to 20 psi, all using nitrogen. Quantification was performed using the following ion transitions: m/z 649.6 > 603.1 for GRh_3_, with a declustering potential (DP) of −50.0 V and collision energy (CE) of −26.0 eV. For the IS, the ion transition was 665.4 > 619.4, with DP at −50.0 V and CE at −30.0 eV.

### 2.8 Method validation

The LC-MS/MS method for quantifying GRh_3_ in rat plasma and tissues was validated according to the guidelines for *Bioanalytical Method Validation* issued by the FDA and the European Medicines Agency (EMEA) (EMA, 2011; FDA, 2018). Considering the liver’s prominent metabolic activity and pronounced matrix effects, it was selected as the representative tissue for methodological validation in this study. The validation parameters encompassed selectivity, carryover, linearity, precision, accuracy, matrix effects, extraction recovery, and sample stability.

#### 2.8.1 Selectivity

Selectivity was assessed based on matrix selectivity (endogenous interference), interference of the GRh_3_ with the IS, and interference of the IS with GRh_3_.

##### 2.8.1.1 Matrix selectivity

This was evaluated using double blank samples (without GRh_3_ and IS) and LLOQ samples prepared from blank matrices of six individual rats. The peak areas for GRh_3_ and IS at their respective retention times in the double blank samples should not exceed 20.0% and 5.0%, respectively, of the corresponding peak areas in the LLOQ samples.

##### 2.8.1.2 Interference of GRh_3_ with IS

Six samples containing only GRh_3_ (ULOQ without IS) were prepared. The average peak area at the IS retention time in these samples should not exceed 5.0% of the average peak area of the IS in standard calibration and QC samples from the same analytical batch.

##### 2.8.1.3 Interference of IS with GRh3

Six samples containing only the IS (QC0) were prepared. The average peak area at GRh_3_ retention time in these samples should not exceed 20.0% of the average peak area of GRh_3_ in LLOQ samples from the same analytical batch.

#### 2.8.2 Carryover

Carryover was evaluated by injecting double blank samples after ULOQ samples (carryover samples). The peak area of GRh_3_ in carryover samples should not exceed 20.0% of the mean peak area in LLOQ samples. Similarly, the peak area of IS in carryover samples should not exceed 5.0% of the mean peak area in standard calibration and QC samples.

#### 2.8.3 Linearity

The calibration curve was constructed by performing linear regression of the GRh_3_-to IS peak area ratios (*y*) against the nominal concentrations of GRh_3_ (*x*), using a 1/*x* ^2^ weighting factor. The measured concentrations at each calibration samples should deviate from the theoretical values by ± 15.0% (±20.0% for LLOQ), and the correlation coefficient (*r*) should be ≥ 0.99. A minimum of six concentration points, including LLOQ and ULOQ, were used to construct the calibration curve.

#### 2.8.4 Precision and accuracy

Precision and accuracy were assessed at four levels (LLOQ QC, LQC, MQC, and HQC), with six replicates per level, prepared independently over three consecutive days. Precision was evaluated by calculating the relative standard deviation (R.S.D%) of the measured concentrations for the replicate samples. Accuracy was assessed by comparing the ratio of the measured to the theoretical concentrations (%). The ratio should fall within 85.0%–115.0% (80.0%–120.0% for LLOQ QC), and the R.S.D% should not exceed 15.0% (20.0% for LLOQ QC).

#### 2.8.5 Extraction recovery and matrix effect

Extraction recovery and matrix effect were evaluated at three levels (LQC, MQC, and HQC), with six replicates. For extraction recovery evaluating, the QC samples prepared for precision and accuracy assessments were used as test samples, while blank plasma and blank tissue homogenate extracts spiked with GRh_3_ and IS served as the basic samples. For matrix effect assessment, basic samples for extraction recovery were used as test samples, and PBS (as a surrogate matrix) spiked with GRh_3_ and IS post-extraction served as the basic samples. The absolute extraction recovery and absolute matrix effect for GRh_3_ and IS should have an R.S.D% not exceeding 15.0%. The IS-normalized extraction recovery and IS-normalized matrix effect should be within 85%–115%, with an R.S.D% not exceeding 15.0%.

#### 2.8.6 Sample stability

Sample stability was assessed under the following conditions: 3 h at room temperature under white light, 30 days at −80°C, and three freeze-thaw cycles (from −80°C to room temperature). This assessment was performed at three concentration levels (LQC, MQC, and HQC), with six replicates. The acceptance criteria are that the relative error (R.E%) between the measured and nominal concentrations should be within ± 15.0%, and the R.S.D% should not exceed 15.0%.

#### 2.8.7 Post-preparation sample stability

Post-preparation stability was assessed at three levels (LQC, MQC, and HQC), with six replicates, under the following conditions: 3 h at room temperature under white light, 12 h in an autosampler at 8°C, and 2 days in a refrigerator at −20°C. The acceptance criteria for this evaluation are consistent with those applied in the sample stability assessments.

#### 2.8.8 Statistical analysis

LC-MS/MS data acquisition and peak integration were performed using Analyst™ software (AB Sciex, version 1.6.3). The concentrations of GRh_3_ in plasma and tissues were calculated based on the calibration curve. PK parameters, including the area under the concentration-time curve (AUC), mean residence times (MRT), elimination half-life (T_1/2_), peak plasma concentration (C_max_), volume of distribution (V_d_), T_max_, and clearance rate (CL) were calculated using Phoenix WinNonlin 8.1 (Certara, New Jersey, United States). Data were presented as mean ± standard deviation (SD) and visualized using GraphPad Prism software (version 9.5.1, San Diego, CA, United States).

## 3 Results and discussion

### 3.1 Selectivity

**FIGURE 1:**
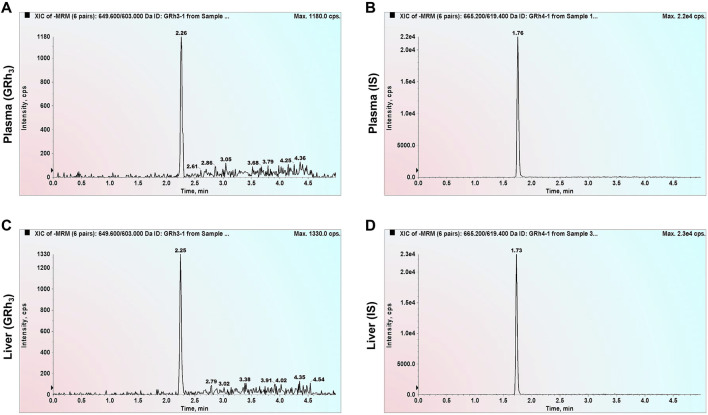
MRM chromatograms of LLOQ samples. **(A)** GRh_3_ in plasma. **(B)** IS in plasma. **(C)** GRh_3_ in liver. **(D)** IS in liver.

**FIGURE 2:**
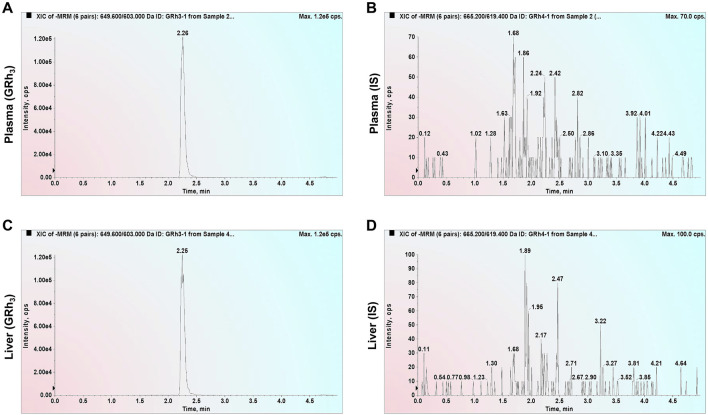
MRM chromatograms of ULOQ without IS samples. **(A)** GRh_3_ in plasma. **(B)** IS in plasma. **(C)** GRh_3_ in liver. **(D)** IS in liver.

**FIGURE 3:**
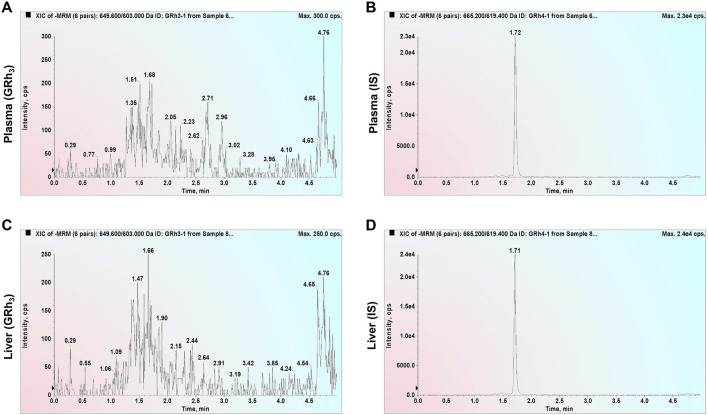
MRM chromatograms of QC0 samples. **(A)** GRh_3_ in plasma. **(B)** IS in plasma. **(C)** GRh_3_ in liver. **(D)** IS in liver.

Double-blank plasma and liver samples were analyzed, showing no detectable GRh_3_ or IS in either matrix. In Figure 1, MRM chromatograms at the LLOQ level for both matrices display well-defined peaks for GRh_3_ and IS, with no evidence of matrix interference. Figure 2 shows MRM chromatograms of ULOQ without IS samples in plasma and liver homogenate, where no IS peaks were detected. Figure 3 presents QC0 samples in the same matrices, confirming the absence of GRh_3_. These findings collectively illustrate that the analytical method is no matrix interference, and mutual non-interference between GRh_3_ and IS. Overall, this method demonstrates excellent selectivity.

### 3.2 Carryover

Carryover samples from plasma and liver were analyzed. Neither GRh_3_ nor GRh_4_ were detected, indicating no carryover interference in this analytical method.

### 3.3 Linearity

**FIGURE 4:**
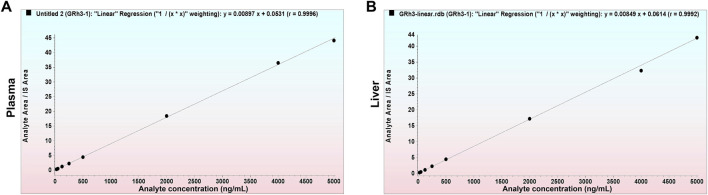
Representative calibration curves. **(A)** GRh_3_ in plasma. **(B)** GRh_3_ in liver.

The deviation between calculated and nominal concentrations of standard calibration samples was within ± 15.0% (±20.0% for LLOQ). Figure 4 presents representative calibration curves for plasma and tissue, while Table 1 summarizes linearity assessment results from three analytical batches for precision and accuracy evaluations. GRh_3_ showed excellent linearity within the concentration range of 25.0–5000 ng/mL, with correlation coefficients (*r*) exceeding 0.99.

**TABLE 1:** Linear parameters for precision and accuracy evaluation batches.

Matrix	Range (ng/mL)	Batch	a (slope)	b (intercept)	*r*
Plasma	25.0–5000	A&P-1	0.008971	0.05312	0.9996
		A&P-2	0.008651	0.05693	0.9989
		A&P-3	0.008748	0.05010	0.9992
Liver	25.0–5000	A&P-1	0.008493	0.06136	0.9992
		A&P-2	0.008812	0.04989	0.9994
		A&P-3	0.007643	0.03514	0.9983

### 3.4 Precision and accuracy

The intra-day and inter-day precision and accuracy results are detailed in Table 2. At four QC levels, the intra-day accuracies for plasma and liver were within 89.3%–112.3% and 85.9%–112.5%, respectively, with intra-day precisions (R.S.D%) being less than 10.7% and 12.8%, respectively. The inter-day accuracies for plasma and liver were within 90.2%–102.0% and 89.3%–105.9%, respectively, with inter-day precisions (R.S.D%) being less than 10.6% and 12.2%, respectively. These results indicate that the method provides excellent precision and accuracy for the quantification of GRh_3_ in plasma and tissue samples.

**TABLE 2:** Intra-day and inter-day accuracy and precision of GRh_3_.

Matrix	Nominal concentration (ng/mL)	Intra-day (*n* = 6)		Inter-day (*n* = 18)	
		Accuracy (%)	Precision R.S.D. (%)	Accuracy (%)	Precision R.S.D. (%)
Plasma	25	89.6–112.3	4.3–10.7	90.2	10.6
	100	90.1–101.1	2.9–8.4	101.4	8.7
	400	93.4–103.2	5.8–6.4	102.0	6.3
	3000	89.3–111.0	3.9–7.0	98.2	8.3
Liver	25	85.9–109.7	6.4–12.8	89.3	12.2
	100	91.6–102.6	7.1–9.9	97.7	7.3
	400	94.0–110.2	4.5–11.0	100.6	8.8
	3000	90.3–112.5	3.7–9.7	105.9	9.2

### 3.5 Extraction recovery and matrix effect

The extraction recovery and matrix effect were assessed using three QC concentrations, as detailed in Table 3. For plasma, GRh_3_ demonstrated an extraction recovery ranging from 93.2% to 97.1% (R.S.D% < 10.0%), whereas the IS exhibited an extraction recovery of 96.7% (R.S.D% = 9.6%). Following IS normalization, the corrected extraction recoveries for GRh_3_ were between 96.4% and 100.4%. In liver tissue, GRh_3_ showed an extraction recovery range of 92.4%–98.7% (R.S.D% < 9.5%), with the IS displaying an extraction recovery of 94.2% (R.S.D% = 11.3%). Post-IS correction, the adjusted extraction recoveries for GRh_3_ were between 98.1% and 104.8%. Regarding matrix effects in plasma, GRh_3_ values spanned 98.7%–103.5% (R.S.D% < 8.2%), while the IS matrix effect was 97.9% (R.S.D% = 6.7%). After IS correction, the adjusted matrix effects for GRh_3_ were between 97.9% and 103.5%. In liver tissue, GRh_3_ matrix effects ranged from 94.3% to 100.3% (R.S.D% < 8.8%), with the IS matrix effect being 98.1% (R.S.D% = 8.3%). Post-IS correction, the adjusted matrix effects for GRh_3_ were between 96.1% and 102.2%.

**TABLE 3:** Extraction recoveries and matrix effects of GRh_3_ and GRh_4_ (IS) in plasma and liver homogenate (*n* = 6).

Matrix	Nominal concentration (ng/mL)	Absolutely				IS normalization	
		Extraction recovery (%)	R.S.D. (%)	Matrix effect (%)	R.S.D. (%)	Extraction recovery (%)	Matrix effect (%)
Plasma	100	93.2	10.0	98.7	8.2	96.4	97.9
	400	95.6	5.8	101.6	6.7	98.9	101.3
	3000	97.1	7.2	103.5	7.6	100.4	103.5
Liver	100	92.4	9.5	100.3	8.8	98.1	102.2
	400	93.6	8.5	94.3	7.6	99.4	96.1
	3000	98.7	6.3	97.6	4.2	104.8	99.5
Plasma-IS	50000	96.7	9.6	97.9	6.7	—	—
Liver-IS	50000	94.2	11.3	98.1	8.3	—	—

### 3.6 Sample stability

Collected samples are not immediately processed for analysis but are temporarily stored at −80°C until needed, at which point they are thawed and brought to room temperature for sample preparation. To ensure the reliability and precision of analytical outcomes, it is crucial to assess the stability of these samples under various conditions. The assessment results, as detailed in Table 4, reveal that plasma and liver homogenate with three QC concentrations maintain their stability under diverse conditions: when kept at room temperature for 3 h (R.E%: −4.3%–5.7%, R.S.D% < 9.2%), stored at −80°C for up to 30 days (R.E%: −6.9% to −4.2%, R.S.D% < 9.7%), and subjected to three freeze-thaw cycles (R.E%: −12.3% to −2.3%, R.S.D% < 9.7%). These findings underscore the robust stability of the samples across all tested conditions, confirming that they meet stringent stability criteria.

**TABLE 4:** Sample stability of GRh_3_ in plasma and liver homogenate (*n* = 6).

Matrix	Nominal concentration (ng/mL)	3 h at room temperature		30 days at refrigerator (−80°C)		Three freeze-thaw cycles	
		Accuracy R.E (%)	Precision R.S.D. (%)	Accuracy R.E (%)	Precision R.S.D. (%)	Accuracy R.E (%)	Precision R.S.D. (%)
Plasma	100	4.5	4.2	−4.2	8.4	−12.3	9.7
	400	−3.0	8.5	−5.4	6.4	−4.1	6.1
	3000	−4.3	7.1	−6.9	8.9	−2.7	8.8
Liver	100	−1.8	9.2	−5.7	9.7	−9.3	7.5
	400	4.4	3.4	−6.9	5.3	−2.6	6.5
	3000	5.7	6.2	−6.8	6.2	−2.3	2.5

### 3.7 Post-preparation sample stability

Post-preparation samples are not immediately analyzed but are instead subjected to periods of storage at room temperature and in an autosampler (8°C). Additionally, in this study, some samples are temporarily stored in a refrigerator at −20°C until analysis. To ensure the reliability and precision of analytical outcomes, it is crucial to assess the stability of post-preparation samples under various conditions: at room temperature, in an autosampler (8°C), and in a refrigerator at −20°C. As detailed in Table 5, post-preparation samples exhibit satisfactory stability when kept at room temperature for 3 h (R.E%: −6.0%–6.6%, R.S.D% < 10.2%), in an autosampler (8°C) for 12 h (R.E%: −8.5% to −2.7%, R.S.D% < 9.0%), and in a refrigerator at −20°C for up to 2 days (R.E%: −8.2% to −4.9%, R.S.D% < 10.1%). These findings confirm that the samples meet the necessary stability criteria, thereby ensuring the validity and accuracy of subsequent analyses. These results underscore the stability of post-preparation samples across all conditions. Consequently, this method ensures the validity and accuracy of subsequent analyses.

**TABLE 5:** Post-preparation sample stability (*n* = 6).

Analytes	Nominal concentration (ng/mL)	3 h at room temperature		12 h at auto-sampler (8°C)		2 days at refrigerator (−20°C)	
		Accuracy R.E (%)	Precision R.S.D. (%)	Accuracy R.E (%)	Precision R.S.D. (%)	Accuracy R.E (%)	Precision R.S.D. (%)
Plasma	100	2.7	3.3	−4.2	4.8	−7.6	8.8
	400	−2.2	7.1	−3.5	7.2	−5.3	7.0
	3000	−6.0	10.2	−2.7	7.3	−8.2	5.9
Liver	100	−2.8	5.0	−8.5	9.0	−8.2	10.1
	400	6.6	4.8	−7.7	7.1	−4.9	6.9
	3000	6.3	3.6	−4.9	3.9	−7.4	5.7

### 3.8 Pharmacokinetics analysis

**FIGURE 5:**
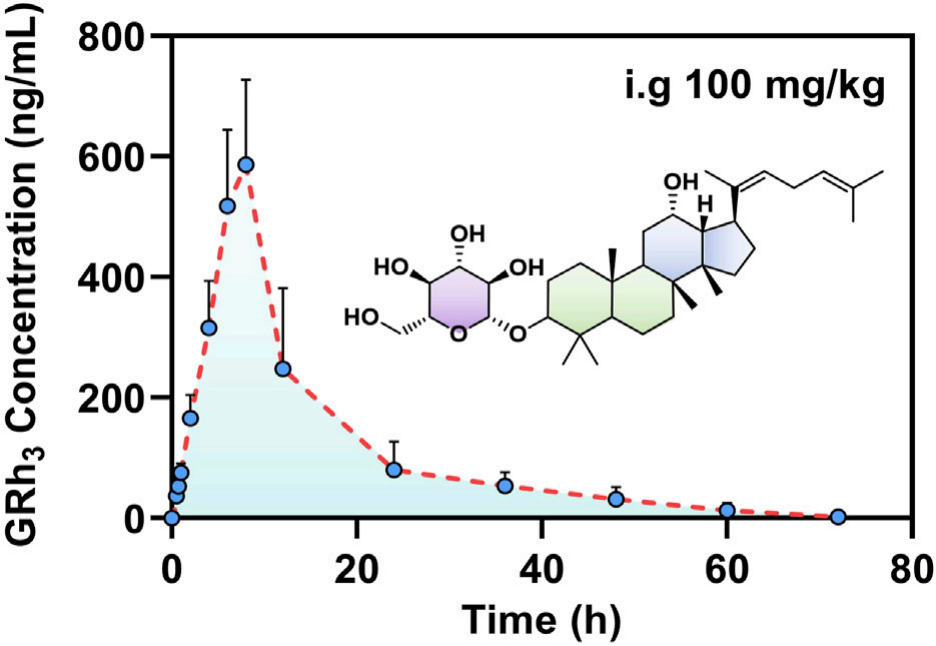
The plasma concentration-time profile of GRh_3_ after oral administration of 100 mg/kg in rats (*n* = 7).

After intragastric administration of GRh_3_, the plasma concentration-time profile is illustrated in Figure 5, while the PK parameters derived from non-compartmental analysis are summarized in Table 6. The findings reveal a T_max_ of 8.0 h for GRh_3_, with a T_1/2_ of 14.7 ± 1.7 h. MRT were calculated as 14.4 ± 2.8 h for MRT_(0-t)_ and 18.5 ± 1.8 h for ${\text{MRT}}_{\left(0-\infty \right)}$ . Moreover, the CL was determined to be 13.0 ± 3.8 L/h/kg, and the V_d_ was estimated at 280.4 ± 109.3 L/kg.

**TABLE 6:** PK parameters of GRh_3_ after oral administration of 100 mg/kg in rats (*n* = 7).

Parameter	Unit	Mean ± SD
AUC_(0–t)_	μg/L·h	7742.9 ± 2441.4
${\text{AUC}}_{\left(0-\infty \right)}$	μg/L·h	8272.9 ± 2348.0
MRT_(0–t)_	h	14.4 ± 2.8
${\text{MRT}}_{\left(0-\infty \right)}$	h	18.5 ± 1.8
T_1/2_	h	14.7 ± 1.7
T_max_	h	8.0 ± 0.0
V_d_	L/kg	280.4 ± 109.3
CL	L/h/kg	13.0 ± 3.8
C_max_	μg/L	586.6 ± 140.5

### 3.9 Tissue distribution study

**FIGURE 6:**
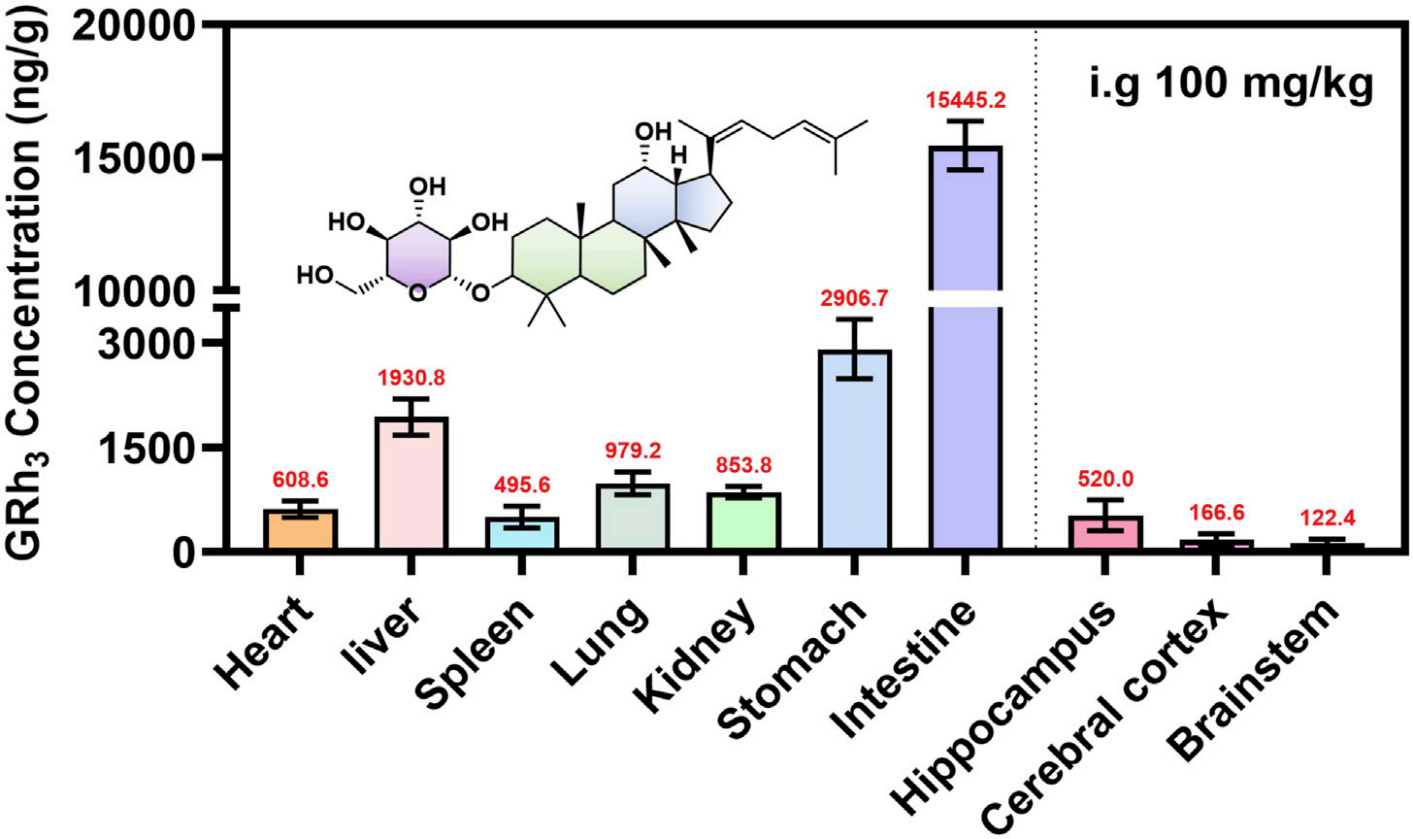
The tissue distribution of GRh_3_ after oral administration of 100 mg/kg in rats (*n* = 7).

To comprehensively characterize the tissue distribution of GRh_3_ in rats, tissues were harvested at T_max_ (8.0 h) post-intragastric administration, and the GRh_3_ content in each tissue was quantitatively determined. The results reveal extensive distribution of GRh_3_ across multiple tissues, with notably higher concentrations in certain organs compared to plasma levels (Figure 6). Specifically, the highest concentrations were observed in the intestines (15445.2 ng/g), followed by the stomach (2906.7 ng/g) and liver (1930.8 ng/g). Remarkably, GRh_3_ demonstrated substantial brain penetration, accumulating prominently in the hippocampus, where concentrations reached up to 520.0 ng/g. These findings highlight the broad tissue distribution and significant brain transmissivity of GRh_3_.

## 4 Discussion

**FIGURE 7:**
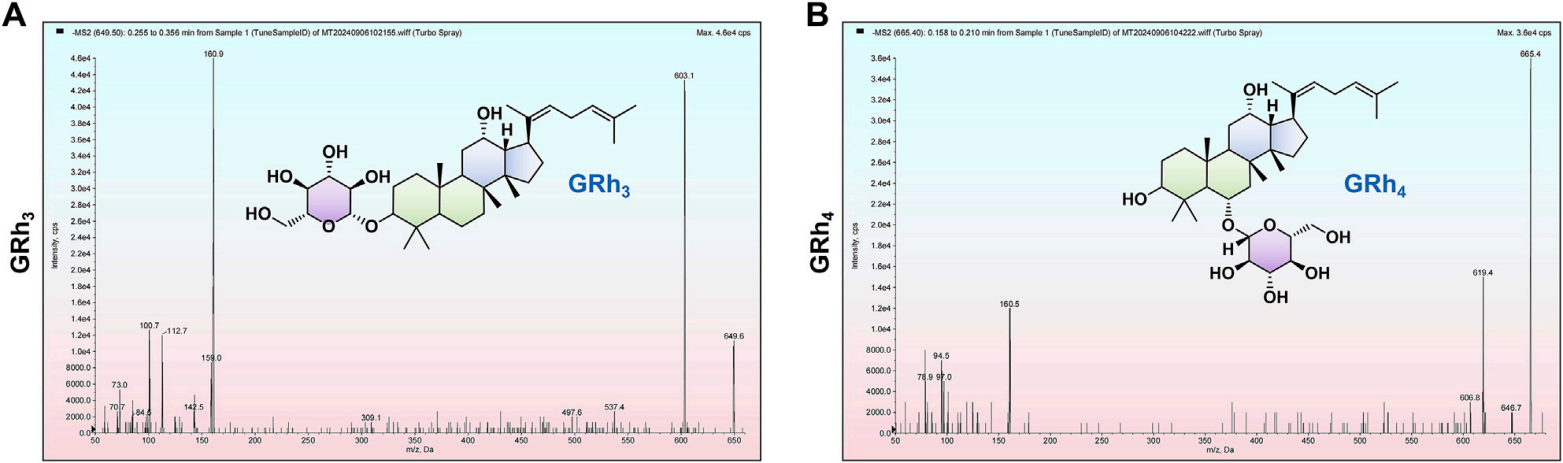
The [M+HCOO]^−^ product ion scans. **(A)** GRh_3_. **(B)** GRh_4_.

This study developed and validated a highly sensitive and selective method based on LC-MS/MS for the precise quantification of GRh_3_ in rat plasma and various tissues. During method development, Q1 full scans revealed that GRh_3_ exhibited the highest intensity in the [M+HCOO]^−^, which directed subsequent product ion scanning (Figure 7A). The two most intense product ions were paired with [M+HCOO]^−^ to establish the MRM quantitative ion transitions. Systematic optimization of key parameters (DP, CE, ion source temperature, spray voltage, and gas settings) maximized signal intensity. The optimization of column selection and mobile phase conditions revealed that the Shimadzu Shim-pack GIST-HP C18 column (2.1 mm × 50 mm, 3 μm), paired with a water (0.1% formic acid)/acetonitrile system, provided optimal chromatographic separation and maximized analytical sensitivity. The ion transition 649.6 > 603.1 was ultimately selected for GRh_3_ quantification based on peak symmetry, sensitivity, and minimal interference. As no isotopically labeled IS for GRh_3_ was available, GRh_4_ was selected as the IS due to its similar properties. The optimization for GRh_4_ followed the same procedure. The GRh_4_ product ion scan was conducted (Figure 7B), and the ion transition 665.5 > 619.4 was chosen for detection.

The PK data demonstrate that GRh_3_ exhibits a “prolonged retention-low clearance” sustained-release profile. The extended time to reach peak plasma concentration (T_max_ = 8.0 h) suggests a delayed absorption phenomenon, which may be attributed to gastrointestinal retention effects or significant first-pass elimination (Omeh et al., 2022; Pond and Tozer, 1984). Consequently, this delays the entry of GRh_3_ into the systemic circulation. From the perspective of elimination half-life and mean residence time, GRh_3_ exhibits relatively high T_1/2_ (14.7 ± 1.7 h), MRT_(0-t)_ (14.4 ± 2.8 h), and ${\text{MRT}}_{\left(0-\infty \right)}$ (18.5 ± 1.8 h). These results suggest that GRh_3_ may undergo tissue redistribution. In terms of apparent volume of distribution and clearance, GRh_3_ exhibited a significantly larger V_d_ (280.4 ± 109.3 L/kg) compared to conventional drugs, suggesting that GRh_3_ may be widely distributed in peripheral tissues (Hoch et al., 2024). Its low CL (13.0 ± 3.8 L/h/kg) was associated with the “reservoir effect” caused by tissue accumulation. This “reservoir effect” reduce the free drug concentration, thereby decreasing systemic clearance and ultimately prolonging the GRh_3_’s T_1/2_ (Eusébio et al., 2024). Although this study is the first to reveal the sustained-release pharmacokinetic characteristics of GRh_3_, some limitations exist. Future research could employ radiolabeling techniques to trace GRh_3_’s tissue distribution pathways or develop population pharmacokinetic models based on transporter gene polymorphisms. These approaches could help optimize the clinical application of GRh_3_, particularly in chronic disease treatment, leveraging its long-acting properties.

Tissue distribution data indicated that the highest concentrations of GRh_3_ in the intestines, followed by the stomach and liver, indicative of a pronounced first-pass effect (Hervieu et al., 2025). These findings are consistent with the high V_d_, prolonged T_1/2_, and extended MRT observed in the PK data. Notably, GRh_3_ also exhibits significant brain distribution, particularly in the hippocampus, where its concentrations reached up to 520.0 ng/g, exceeding levels observed in the spleen (495.6 ng/g). The classical BBB theory posits that compounds with high lipophilicity and molecular weight below 500 Da can passively diffuse through the BBB (Xie et al., 2019). However, tissue distribution study has shown orally administered GRh_3_ accumulates in rat brain parenchyma despite its molecular weight of 604 Da, which deviates significantly from the BBB permeability criteria.

This finding suggests that passive diffusion alone cannot fully explain the observed central nervous system (CNS) distribution of GRh_3_, implying the involvement of active transporter-mediated mechanisms. The BBB expresses a diverse array of transporters that regulate the movement of substances into and out of the brain. These transporters are broadly categorized into influx and efflux systems (Parvez et al., 2023; Ronaldson and Davis, 2015). Influx transporters, including organic anion transporting polypeptides (OATPs), organic cation transporters (OCTs), monocarboxylate transporters (MCTs), L-type amino acid transporter 1 (LAT1), and glucose transporters (GLUTs), facilitate the uptake of nutrients and drugs from the bloodstream into the brain (Parvez et al., 2023; Tamai and Tsuji, 2000). Conversely, efflux transporters such as P-glycoprotein (P-gp), breast cancer resistance protein (BCRP), and multidrug resistance-associated proteins (MRPs) actively expel substances from the brain, helping to maintain CNS homeostasis (Cox et al., 2023; Parvez et al., 2023). Previous studies have reported that GRb_1_ can cross the BBB through interactions with GLUT1 (Wang et al., 2018), and that GRg_3_ has been shown to act as a P-gp inhibitor at the BBB (Xu et al., 2021). Ginsenosides share a similar structural backbone, and GRh_3_ is likely to retain critical molecular characteristics that support interactions with transporters in a manner similar to its analogs, potentially enabling its transport across the BBB into the brain parenchyma.

In addition to potential active transport, the integrity of the BBB may also contribute to GRh_3_’s accumulation in the CNS. Our previous *in vitro* studies demonstrated that GRh_3_ induces oxidative stress and neuronal apoptosis via the IP3R-Ca^2+^/NOX2/NF-κB pathway (Wang et al., 2025). Both oxidative stress and inflammation have been implicated in BBB disruption (Fang et al., 2024; Gao et al., 2023), suggesting that GRh_3_ may impair BBB function and thereby facilitate its own entry into the brain. This is supported by the notably high concentration of GRh_3_ in the hippocampus, a brain region highly susceptible to oxidative damage and neuroinflammation. To verify this possibility, future studies should assess BBB integrity following GRh_3_ administration using classical methods such as Evans Blue dye extravasation and immunohistochemical detection of tight junction proteins, including Claudin-5 and Occludin. Overall, elucidating both active transport mechanisms and structural BBB alterations will be critical for fully understanding the brain pharmacokinetics and potential neurotoxicity of GRh_3_.

## 5 Conclusion

This study developed and validated a LC-MS/MS method for detecting GRh_3_ concentrations in rat plasma and tissues. Subsequently, the PK result indicated that oral administration of GRh_3_ exhibited a prolonged T_max_ (8.0 h) and an extended T_1/2_ (14.7 ± 1.7 h), indicating relatively slow absorption and excretion processes. The tissue distribution data showed that GRh_3_ significantly accumulated in the intestine, stomach, and liver, and was also able to penetrate the BBB to accumulate in brain tissues, particularly in the hippocampus. However, the mechanisms underlying its BBB penetration remain to be further investigated. This study provides essential support for the further research and development of GRh_3_.

## Data Availability

The original contributions presented in the study are included in the article/supplementary material, further inquiries can be directed to the corresponding authors.
